# Correspondence

**Published:** 1901-01

**Authors:** S. H. Swain

**Affiliations:** Decatur, Ill.


### CORRESPONDENCE.

DECATUR, ILL., January 1, 1901.

EDITOR JOURNAL OF COMPARATIVE MEDICINE, ETC.:

IN the November number of the *American Veterinary Review*, p. 636, there appears an intellectual monstrosity painted by the pencil of Dr. James Robertson, D.V.S., member of the Illinois State Board of Veterinary Examiners, wherein the doctor seeks to extricate himself from a very painful and peculiar dilemma in which he is placed by a published arraignment of the board's official conduct by the Committee of the Illinois Veterinary Medical and Surgical Association.

This committee did not shirk its responsibility nor deal in terms of maudlin sentiment, but, as honest and earnest men, inspired by a high sense of duty to their profession, proceeded at once to investigate a state of facts so revolting that a solemn sense of duty bade them decide to cauterize and clean out this foul and offensive excrescence calling itself "The State Board of Examiners." We have no option, but are driven to this duty because of dereliction of duty and flagrant violation of law amounting almost to malfeasance in office. From the doctor's affected, inflated, sonorous, and high-sounding style in defensive reply to our published report the profession may readily gauge the intellectual and logical force of even this guileless graduate to successfully defend himself and his board when "weighed in the balance and found wanting." The doctor's defence is a sad surprise to us, coming from a source so eminent, as it furnishes us neither literary flowers nor any sort of fruit. It actually reminds us of Grover Cleveland's report to Mrs. Henry Ward Beecher with respect to his moral conduct, which was cunningly constructed, but when rightly reduced to the last analysis it was found to be one-half alibi and the other half confession. But, if I may presume to further characterize the doctor's contribution, it seems to be entirely innocent of ideas, of clearness, conciseness, and classical culture. It is weak and wandering, murky and muddled; it is in the right road only when crossing it, and no sane man can decide what he is aiming at, which side it is on, or which way it is going. It reminds us of the Irishman who followed the winding trail of a serpent until it entered its den, and then in bewilderment Pat declared that

> "It so wriggled in and wriggled out
> That it leaves the observer much in doubt
> Whether the snake that made the track
> Was coming out or going back."

The truth will appear to all who read the doctor's defence that his sympathetic heart and his avarice are entirely too much for his judicial head and sense of moral responsibility. He depicts the old, illiterate moss-back practitioner coming before his board, ignorant of even the rudimentary principles of the science or art of our profession, and yet the board arms the incompetent with a license to prey upon the public and inflict infernal torture upon dumb beasts because the applicant is poor in purse, burdened with children, and because he has the fee. Sentiment and twenty silver dollars too often sway the effeminate and unworthy official, but the stalwart soul feels that duty is the grandest word in this world. If delegated to perform a sacred duty, sentiment is utterly out of place. If placed upon the picket line facing the enemy, the officer has a plain and positive duty to perform, and against the law laid down for his guidance; and he is held to be criminal if he exercises any option or is influenced in the least by sentiment or sympathy or silver as to whom he shall wrongfully let pass through his lines, to the injury of the confiding body of nobler men behind him. A man who is thus honored with an important position of *trust* must not *prostitute it!* It is criminal. The doctor does not deny that

ARMY MEDICAL LIBRARY
WASHINGTON, D. C. U. S. A.

he ever rejected an applicant. *Think of it!* And because our committee complain of this courage there comes creeping out from under the murky cloud of his article a seething, hissing sneer at us as "non-graduated." Is that his only or his best defence against our just and righteous arraignment of his flagrant violation of the law?

Now, Mr. Editor, upon this point which the doctor brings forward let me suggest to him a little common sense. I say only a little, as his head is evidently incapable of holding very much. Whenever a diploma is conferred by a veterinary college upon a man of studious habits, of sterling integrity, and sound common sense, and when that diploma has been well and worthily won, and is thus an actual evidence of the owner's education in his profession, *then* and only then can I consent to uncover my head before a collegian with his sheepskin. But when a diploma is prostituted to unworthy work; when it is regarded and flaunted before our face not as a *help*, but as the *whole thing;* when it is used as a cloak to cover up absolute incompetency, as in the case of his eminence at whom this article is aimed, then a diploma becomes a dishonor, a degradation, and an actual curse. It dishonors alike him who has not sense or culture enough to worthily hold it and the college and profession that conferred it. "I hope upon this point I am clearly understood."

And, sir, permit me to say, as respects myself, that "the crime of being a non-graduate" I shall attempt neither to palliate nor deny. In simple ignorance I would be your equal not to know that I am the man at whom you aim this harmless blow. But, in order to oblige you, I may say I wish the fact known among all men that I am a non-graduate; my place and position in my profession enable me to be thus independent of artificial aid. So let it be placarded on our banner as I walk the Golden Streets of the New Jerusalem with the great body of the very best and brainiest men of our profession, and I wish it heralded all over hell, where all dishonest doctors, incompetents, and quacks are sure to hear it. But, my dear sir, wherever you hold aloft your diploma we will run up over it the scalpel and the skeleton. These I prefer shall suggest the source of my proficiency in the profession, for I have yet to find a good veterinary surgeon who is a poor anatomist, nor have I ever seen a poor surgeon who is a first-class anatomist. It makes no difference where a man studies, whether in the woods or in a cabin, or in a castle or a college; but to be a physician and a surgeon he must *study* and be blessed with a sound mind in a sound body, with an honest heart and a cool and clear head, for genius never stamps her image on common clay. I deprecate and denounce the doctor's effort to disparage the non-graduate, for a man may be vastly more learned and efficient without a diploma than others who have them. Does he not know that when the great Daniel Webster received his diploma he tore it in shreds and said, "My energy and industry may make a man of me, but this miserable parchment never shall"? Elihu Burritt, the learned blacksmith, acquired thirty-two different languages while working with his book before him at the forge. But do not understand me that every blacksmith has the brain of Elihu Burritt. Robert Burns and William Shakespeare, who have swayed the world with their sublime poetry for two hundred years, and Abraham Lincoln—in every sense the equal of Moses and Solomon—were not only non-graduates, but they never even went to school. I cite these few from among the millions for the purpose, if possible, of shedding a ray of light into the dim and dark understanding of the doctor's murky mind. Let it be understood that no one can honor educated collegians more than I, but they have their peers among the great army of able men without diplomas. We are all students and workers alike in one cherished, common cause; and the distinctions such as the doctor assumes are not only injurious and odious, but can emanate only from a very little man with a very shallow mind.

Thus far we have curried the doctor and his examining board with the softest silk, but we must now come to something more specific, which fully sustains the worst we have said.

In the city of Mattoon, Ill., at the semi-annual meeting of the Illinois Veterinary Medical and Surgical Association, in the month of August, 1900, and before a well-attended meeting, there being present visitors of eminence from the Illinois State Veterinary Medical Association, appeared an applicant for membership in our Association who had successfully passed the requisite examination before the State Board of Examiners. He appeared before our committee, and we proceeded to give him the usual examination. The questions put to him in anatomy, physiology, pathology, surgery, materia medica, and obstetrics were primary and of the simplest sort, and to our utter amazement the man failed to answer a single question. When asked to locate the inferior maxilla he was unable to do so, nor could he give the number of incisor or molar teeth or locate the tarsus or metacarpal bones. He could not locate any section of the vertebra or even the coccix. He was ignorant of the number of costals. He knew absolutely nothing of anatomy, physiology, pathology, materia medica, symptomatology, therapeutics, or surgery, and yet this man was graduated by the State Board of Veterinary Medical and Surgical Examiners to practise veterinary medicine and surgery in the State of Illinois! After being rejected by our committee he came before and was questioned by the Association, including Dr. W. J. Martin and Dr. N. P. Whitmore, of the Illinois State Veterinary Medical Association, and numerous others, to whom he declared that he had passed the requisite examination before the State Board and that he had their permit to practise in his pocket. He stated the board asked him the following questions: Are you married? How many children? What are you worth? How long have you practised? If he could castrate and spay, and was questioned principally on these latter, and he declared it as his belief that the board sought to secure his methods of spaying and castration. We had some doubts on these points, but finally he convinced us. Their first demand on him, he said, was the fee of twenty dollars. He asked that in case of failure in passing if they would refund the fee. They said, "No, but that need not disturb him, because he would pass beyond all question," and *he passed.*

I will cite only one other case from quite a number who passed this brilliant and benevolent Board of State Examiners. A gentleman who was demented and detained in the insane asylum was let out on parole, and while in the bold flights of an extravagant fancy he conceived the idea of taking out a license and entering upon the practice of veterinary medicine and surgery. He went before the State Board of Veterinary Medical Examiners and was vigorously opposed by Dr. Martin and others, but he furnished the fee of twenty dollars and passed the board successfully. Now I would respectfully appeal to the profession at large if this committee was not justified in our published criticism of the board's official conduct and in asking for its removal from office; and to the doctor himself let me say, in all kindness, that although a non-graduate I hold myself equal to meet with overwhelming facts anything further you may care to offer, and for the present will only add—

"Lay on Macduff, and damned be he who first cries hold, enough."

S. H. SWAIN, V.S.

